# Effects of atorvastatin on plasma matrix metalloproteinase-9 concentration after glial tumor resection; a randomized, double blind, placebo controlled trial

**DOI:** 10.1186/2008-2231-22-10

**Published:** 2014-01-07

**Authors:** Niayesh Mohebbi, Alireza Khoshnevisan, Soheil Naderi, Sina Abdollahzade, Jamshid Salamzadeh, Mohammadreza Javadi, Mojtaba Mojtahedzadeh, Kheirollah Gholami

**Affiliations:** 1 Department of Clinical Pharmacy, Faculty of Pharmacy, Tehran University of Medical Sciences, Tehran, Iran; 2 Department of Neurosurgery, Tehran University of Medical Sciences, Tehran, Iran; 3 Department of Clinical Pharmacy, Shahid Beheshti University of Medical Sciences, Tehran, Iran

**Keywords:** Atorvastatin, Matrix metalloproteinase-9, Neuroprotective effects, Brain injuries, Glial tumor resection

## Abstract

**Background:**

Neurosurgical procedures such as craniotomy and brain tumor resection could potentially lead to unavoidable cerebral injuries. Matrix metalloproteinase-9 (MMP-9) is up-regulated in neurological injuries. Statins have been suggested to reduce MMP- 9 level and lead to neuroprotection. Atorvastatin preoperatively administered to evaluate its neuroprotective effects and outcome assessment in neurosurgical-induced brain injuries after glial tumor resection. In this prospective, randomized, double-blind, placebo-controlled trial, 42 patients undergoing glial tumor surgery randomly received 40 mg atorvastatin or placebo twice daily from seven days prior to operation and continued for a 3 weeks period. Plasma MMP-9 concentration measured 4 times, immediately before starting atorvastatin or placebo, immediately before surgery, 24 hours and two weeks after the surgery. Karnofsky performance score was assessed before first dose of atorvastatin as a baseline and 2 months after the surgery.

**Results:**

Karnofsky performance scale after surgery raised significantly more in Atorvastatin group (11.43 +/- 10.62 vs. 4.00 +/- 8.21) (p = 0.03). Atorvastatin did not significantly reduce MMP-9 plasma concentration 24 hours after surgery in comparison to placebo. No statistical significance detected regarding length of hospital stay among the groups. Significant reduction in MMP-9 plasma concentration was recorded in atorvastatin group two weeks after surgery (p = 0.048).

**Conclusions:**

Significant statistical differences detected with atorvastatin group regarding MMP-9 plasma concentration, clinical outcome and Karnofsky performance score. Consequently, atorvastatin use may lead to better outcome after neurosurgical procedures.

## Introduction

Neurosurgical procedures could be complicated by a diverse spectrum of factors, such as direct trauma, hemorrhage, brain retraction, and electrocautery with subsequent morbidity and mortality. Blood–brain barrier (BBB) disruption and brain edema is one of the proposed causalities [1,2]. Discovering novel neuro-protective agents and suggesting pre-operative regimen can result in better outcome after brain surgeries. The matrix metalloproteinases (MMPs) are enzymes that play important roles in physiologic and pathophysiologic processes. These enzymes’ levels mostly are not detectable, and increase in response to transcriptional regulators [3,4]. MMP-9 (Gelatinase B) is up-regulated in neurological injuries such as cerebral ischemia, BBB disruption, edema formation, and hemorrhagic transformation [5-8]. Different explanations provided to clarify relation of this enzyme and neurotoxicity [9-11]. In particular MMP-9 level rises in acute neurological injuries, so this enzyme proposed to be closely related to neurological impairments [3,12,13]. On the other hand, blockade of MMP-9 activity have shown to be neuroprotective, and appropriate for the treatment of acute brain injuries [14-16]. Experimental studies suggest that preoperative inhibition of MMP-9 reduce brain injuries following neurosurgical procedures [1,2].

Statins not only lower cholesterol effectively [17] but also have other properties called “pleiotropic effects.” Several clinical trials have indicated that statins suppress inflammatory reactions, recover neurological injuries, decrease the incidence of ischemic stroke, improve endothelial function, inhibit platelet activation, control vasospasm following subarachnoid hemorrhage, have neuroprotective activity in spinal cord injury, and prevent progression of Alzheimer’s disease [18-30]. There are published documents claiming that statins decrease MMP-9 levels [30-32]. Many of these evidences are related to atorvastatin, a potent member of this family. This study was designed to evaluate atorvastatin effects on plasma MMP-9 concentration in neurosurgical- induced brain injuries after glial tumor resection. To minimize confounding factors Patients with gliomas grade 1, 2 and 3 which pathologically do not increase MMP-9 plasma levels substantially were entered the study and patients with Glioblastomamultiforme (GBM) were excluded.

## Methods

This is a randomized, double blind, placebo-controlled prospective clinical trial performed in neurosurgery ward of a tertiary care Hospital from March 2011 to December 2012. The protocol was approved by the ethics committee of Tehran University of Medical Sciences (TUMS). This clinical trial was registered at Iranian Registry of Clinical Trial (IRCT) with the registration ID of “IRCT201203039196N1”. For detecting any significant changes of MMP-9 concentration as main endpoint a sample size was calculated to be 21 patients at each group. Standard deviation for plasma MMP9 in human was supposed 23 ng/ml and 20 ng/ml was accepted as significant rise in MMP9 level, with α = 0.05 and ß = 0.20. [n = 2(Z1- α/2+ Z1-β)2 S2/d2, i.e. n = 2(1.96 + 0.84)2 × 232/202, n = 21] Patients undergoing elective surgery as therapeutic approach for tumor resection with diagnosis of glial tumor based on radiographic findings were included in this study. Patients excluded from the study with any atorvastatin contraindications such as, atorvastatin sensitivity, active hepatic disease, pregnancy and lactation; elevated liver enzymes; history of serious adverse reaction induced by atorvastatin; severe renal or hepatic failure; concurrent infectious disease; use of atorvastatin or other statins before the study; pathology report of other type of tumors instead of glioma grade 1,2 and 3; serious interaction between patients medications and atorvastatin; MAP < 70 mmHg; Na < 130 mEq/L; Hematocrit < 28%.

After obtaining informed consent, using permuted-block randomization patients were randomly assigned to atorvastatin or placebo group. Patients received atorvastatin or placebo 40 mg oral tablets, twice daily (totally 80 mg) for 3 weeks, from one week before to 2 weeks after surgery. Standard therapeutic regimen and supportive care, such as antibiotic prophylaxis, seizure prophylaxis, fluid therapy, and gastro intestinal ulcer prophylaxis have been used for both groups. This standard therapeutic regimen was including cefazolin as antibiotic prophylaxis, phenytoin for seizure prophylaxis, appropriate fluid therapy with normal saline, ranitidine as gastrointestinal ulcer prophylaxis, and dexamethasone for controlling brain edema after surgery.

At the beginning of the study, patients’ demographic data such as age, sex, weight, comorbidity (having any concurrent disease other than glial tumor), history of hypertension, drug history, and neurological exam were collected on pre-designed questionnaires. Laboratory data recorded at baseline, were as follow: Fasting blood sugar (FBS), creatinin (Cr), blood urea nitrogen (BUN), white blood cell count (WBC count), hemoglobin, erythrocyte sedimentation rate (ESR), C-reactive protein (CRP), liver function tests (LFTs), lipid profile (including total cholesterol, LDL Cholesterol, HDL Cholesterol, and triglycerides). For detection of plasma MMP-9 concentration blood samples collected 4 times from each patient: immediately before starting atorvastatin or placebo, immediately before surgery, 24 hours and two weeks after the surgery. The plasma MMP-9 measured by enzyme-linked immunosorbent assay (ELISA) kits, Quantikine®, manufactured by R&D Systems, United States of America. Based on the kit catalog recommendations blood samples carried with heparinized tubes on ice. To separate plasma, blood centrifuged for 15 minutes at 1000 × g within 30 minutes of collection. Following the above an additional centrifugation step of the plasma at 10,000 × g for 10 minutes at 2 - 8°C performed for complete platelet removal, then all samples stored at -80°C till the assay day. This kit uses the quantitative sandwich enzyme immunoassay technique to assay MMP-9 levels. A monoclonal antibody specific for MMP-9 has been pre-coated on a microplate. All Standard samples and study samples are pipetted into the wells, and MMP-9 is bound by the immobilized antibody. Following washing away unbound substances, an enzyme-linked polyclonal antibody specific for MMP-9 is added to the wells. After a wash to eliminate unbound antibody-enzyme reagent, a substrate solution is added to the wells and color develops in proportion to the quantity of MMP-9 bound in the primary step. The color expansion is stopped and the intensity of the color is measured.

Clinical outcome were assessed with Karnofsky performance score at the baseline (one week before surgery) and 2 months after the surgery by the same examiner. Type and grade of tumors recorded after pathology report. Hospital stay defined as number of nights patient spent at the hospital. Our patients were closely observed for atorvastatin adverse effects, by interviewing patients during hospitalization and also after discharge in the clinic. Baseline and LFTs was measured in all patients and recheck CPK in any patient with symptoms suggestive of myopathy and also repeat transaminase levels when clinically indicated thereafter was about to be done. High rate reported adverse reactions such as diarrhea, arthralgia, limb pain, myalgia, muscle spasms, musculoskeletal pain, insomnia, nausea, and dyspepsia was asked from each patient specifically.

Statistical analyses were executed with the Statistical Package for Social Sciences (SPSS version 17; SPSS Inc., Chicago, IL, USA) and significance level was defined as p-value less than 0.05. The normal distribution of quantitative data was assessed with one sample Kolmogorov-Smirnov test. Data with a normal distribution were expressed as means ± standard deviations (SDs) and student *t*-test as well as paired *t*-test was used to compare quantitative variables between or within groups, respectively. The Mann–Whitney *U* test and Wilcoxon test were applied to compare non-normally distributed data (expressed as median (inter quartile range) to assess differences between and within groups, respectively. Regarding qualitative variables (categorical data), the chi-square or Fisher's exact test were performed.

## Results

Forty- two patients, (25 males and 17 females) randomly assigned to 21 in atorvastatin group and 21 in placebo group. Patients’ demographic data is presented in Table 1.

**Table 1:** Distribution of the baseline demographic and medical characteristics of patients in atorvastatin and placebo groups

**Parameter**	**Atorvastatin group**	**Placebo group**	**P value**
**Sex**			0.12
Male	15	10	
Female	6	11	
**Age**	53.62 ± 15	40.43 ± 13.09	0.006(95% CI: - 22.31- -4.08)
**Weight**	75.95 ± 12.35	72.43 ± 16.63	0.44(95% CI: - 12.66- 5.61)
**Comorbidity**	14	8	0.06
**Hypertension**	8	3	0.08
**Concurrent drug therapy**	14	8	0.06
**FBS**	94.57 ± 17.72	94.43 ± 24.16	0.98(95% CI: -13.36 – 13.07)
**Cr**	0.93 ± 0.19	0.81 ± 0.15	0.03 (95% CI: -0.23 – -0.01)
**BUN**	19.90 ± 6.36	15.48 ± 5.44	0.02(95% CI: -8.12 – -074)
**WBC count**	7400.48 ± 2857.89	7968.57 ± 379.129	0.98
**ESR**	20.24 ± 8.54	23.00 ± 10.29	0.35(95% CI: -3.13 – 8.66)
**CRP**	10.52 ± 10.96	11.62 ± 10.28	0.50
**Total cholesterol**	198.86 ± 33.45	173.52 ± 31.88	0.006
**LDL cholesterol**	129.62 ± 30.69	107.76 ± 31.73	0.009
**Triglycerides**	153.14 ± 50.58	173.52 ± 31.88	0.04
**Plasma MMP-9 concentration**	256.24 ± 279.81	130.95 ± 78.50	0.33
**Karnofsky performance scale**	82.38 ± 17.29	76.67 ± 19.32	0.25
**Grade of tumor**			0.80
Grade 1	4	3	
Grade 2	15	15	
Grade 3	2	3	
**Type of tumor**			0.73
Ependymoma grade 1	0	1	
Ependymoma grade 2	4	2	
Pilomyxoid astrocytoma	0	2	
Astrocytoma grade 2	5	7	
Oligodendroglioma	3	4	
Ganglioglioma	3	0	
Anaplastic astrocytoma	2	1	
Anaplastic oligodendroglioma	0	2	

Patients mean ± SD age were 53.62 ± 15 years for atorvastatin group, and 40.43 ± 13.09 years in placebo group (p = 0.006, 95% CI: - 22.31- -4.08).

There were no statistically significant difference in regard to baseline parameters such as sex, weight, comorbidity (11 patients with hypertension [26%], 5 patients with ischemic heart disease [12%], 2 patients with gastrointestinal diseases [4.7%], one with lumbar herniated disc [2.4%], a patient with inherited neurofibromyalgia[2.4%], a patient with cataract [2.4%], and one patient with glaucoma [2.4%]), hypertension, concurrent drug therapy, and laboratory data such as FBS, Cr, BUN, WBC count, ESR, CRP (Table 1). The drug history of each patient obtained carefully, and there was no significant difference among the groups regarding medications. Considering drugs that may affect level of MMP-9 just 6 (3 patients in atorvastatin and 3 in placebo group) patients took low doses of aspirin (81 mg) to suppress platelet function. Dexamethasone as a part of standard post-operative protocol administered to all of the patients, 8 mg three times daily.

There were significant differences between baseline lipid profile (total cholesterol (p = 0.006), LDL Cholesterol (p = 0.009), and triglycerides (p = 0.04)) of the study groups. Lipid levels were higher in atorvastatin group; however, in both groups patients were not categorized as hyperlipidemic (Table 1).

Baseline plasma MMP-9 concentration, Karnofsky performance score, type of tumor, and grade of tumor among the groups were not significantly different (Table 1). Pathologic reports indicated grade 1, 2, 3 of glial tumor including, ependymoma grade 1 and 2, pilomyxoid astrocytoma, grade 2 astrocytoma, oligodendroglioma, ganglioglioma, anaplastic astrocytoma, and anaplastic oligodendroglioma.

Comparison of Karnofsky performance scale before and 2 months after surgery showed no significant difference among the groups (p = 0.07), however by further exploration of patients leading to exclusion of just one patient as outlier, significant difference showed up (p = 0.03). Atorvastatin group showed significantly better improvement of Karnofsky performance scale after surgery (11.43 ± 10.62 vs. 4.00 ± 8.21).

MMP-9 plasma level changes, immediately before surgery and 24 hours after surgery was not significantly different among the groups (p = 0.38).

In within group statistical analysis Karnofsky performance score showed significant increase in both atorvastatin (P = 0.001) and placebo (P = 0.03) groups. However, as it is depicted in Table 2 more progression was seen in atorvastatin group. MMP-9 plasma concentration levels increased significantly 24 hours after surgery in placebo group (P = 0.02). Nevertheless, there was no significant rise in MMP-9 levels in the atorvastatin group (P = 0.42). Comparison of MMP-9 plasma levels before surgery and 2 weeks after surgery revealed no significant changes in any of the groups, however there were an increasing trend in the placebo group and a decreasing trend in the atorvastatin group (Table 2).

**Table 2:** Mean ± SD of outcome indicators in atorvastatin (drug) and placebo group

**Variable**	**Plasma MMP-9 concentration before surgery**	**Plasma MMP-9 concentration 24 hours after surgery**	**Plasma MMP-9 concentration 2 weeks after surgery**	**Karnofsky performance scale before surgery**	**Karnofsky performance scale 2 months after surgery**
**Drug group**	190.14 ± 197.74	236.38 ±225.08	164.95 ± 126.68	82.38 ± 17.29	93.81 ± 9.73
**Placebo group**	130.19 ± 83.04	184.81 ± 107.04	180.81 ± 115.93	76.67 ± 19.32	82.86 ± 14.88

Comparison of MMP -9 plasma level changes just before surgery with 2 weeks after surgery demonstrated significant difference among the groups (p = 0.048). Increase in MMP-9 levels 2 weeks after surgery was less in atorvastatin group. Atorvastatin and placebo groups MMP-9 plasma level changes between the first week of drug therapy and before surgery (1^st^ and 2^nd^ measurements), and changes between 3^rd^ and 4^th^ measurements indicated no significant difference. Likewise, comparison of ESR and CRP measurements before and after surgery did not indicate any significance in response to atorvastatin treatment.

Length of hospital stay was slightly in favor of atorvastatin group, but not to a significant degree (8.52 ± 6.77 days for drug group, and 11.29 ± 11.50 days for placebo group). There was no significant relation between age and Karnofsky performance score, MMP9 level changes, or hospital stay in any of the groups. BUN and Cr level changes were not significantly different between two groups. No adverse drug reaction reported by atorvastatin group.

## Discussion

Neurosurgical procedures can induce serious neurological injuries [1,2]. Different medications such as diuretics, osmotic agents, and corticosteroids are suggested to decrease ameliorating brain edema induced by operation, but there is no clinical study in this regard [33]. Immunohistochemical evidences specified that MMP-9 increases surrounding surgical induced brain injury areas [2]. Two experimental studies have demonstrated that MMP inhibitors preserve the BBB, attenuate brain edema, and act as a neuroprotectant after brain surgery [1,2].

There is some evidence that suggesting statins decrease MMP-9 levels [30-32]. Various retrospective and prospective clinical trials, meta-analysis, and researches evaluated pleiotropic effects of statins on surgical outcome. In cardiac, vascular, or non cardiovascular operations, irrespective to the type of surgery, positive effects and reduction of undesirable postoperative outcomes, such as mortality, morbidity, length of hospital and ICU stay have been reported fallowing statin therapy [34-38]. Neuro-protective effects of statins were established in many studies [19-30].

In this prospective, randomized, double blind, controlled trial, 42 patients underwent glial tumor surgery, atorvastatin 40 mg or placebo used two times daily. Since most of the studies use full dose of statins to evaluate surgical induced injuries, 80 mg atorvastatin as a full dose is used in this study. Results demonstrated significant reduction in MMP-9 plasma level in atorvastatin patients after two weeks. Regarding clinical endpoint (Karnofsky performance scale at 2 months after surgery), better outcome observed in atorvastatin group. Length of hospital stay was less in patients who had received atorvastatin, but not to a statistical significant degree.

Plasma MMP9 changes followed the anticipated pattern. No significant change observed in the baseline measurement and second measurement (immediately before surgery), since there was no neuronal injury happened before surgery. Plasma MMP9 increased 24 hours after surgery as expected confirming the hypothesis of MMP9 surge after surgery. There was decrease in the MMP9 plasma concentration 2 weeks after surgery indicating improvement in patients. Between groups, statistical analyses demonstrated that atorvastatin group had significantly more reduction in MMP9 level after two weeks in comparison with placebo.

Within group statistical analyses revealed that atorvastatin diminished MMP-9 peak 24 hours after surgery. This outcome improvement was also confirmed by better neurological outcome as assessed with Karnofsky performance score in atorvastatin group. Despite no significant change in MMP-9 plasma concentration 2 weeks after surgery in within group statistical analyses, mean level increased in placebo group and decreased in atorvastatin group. All these findings indicate beneficial effects of atorvastatin in these patients.

Anti-inflammatory effects and MMP9 suppression appear to be irrelevant to concomitant medications. Although age of two patient groups was significantly different, there was no significant relation between age and outcome variables (i.e. karnofsky performance score, MMP9 level changes, or hospital stay).

Atorvastatin administration was well tolerated during the study period. No adverse drug reaction detected same as other studies on statins [37,38]. Acute kidney injuries were recently reported with high doses of statins [39-42]; therefore renal laboratory values were carefully monitored in this study. There were no significant changes in renal laboratory values between two groups. There is even a retrospective cohort of 98,939 patients who underwent various operations including; major open abdominal, cardiac, thoracic, or vascular procedure between 2000 and 2010 claims that statin use before surgery reduce risk of postoperative acute kidney injury [42]. Preoperative statin therapy decreased the need for postoperative renal replacement therapy [34].

Study had some limitations due to complexity of neurologic, physiologic and pathologic of human brain. Due to diversity of functioning regions of brain each glioma can cause different symptoms and debilities in different functioning parts of human brain within and or between different groups of patients pre and postoperatively. Due to complexity of circuits of brain (mostly unknown) different people suffer from different disabilities even when the glioma is operated on, in same area of brain; different surgical approaches caused different complications that could have been misinterpreted as effect of drug while being merely surgical.

## Conclusion

Pre-operative atorvastatin administration resulted in reduction of MMP-9 level 2 weeks postoperatively and improvement of Karnofsky performance scale 2 month after surgery. Larger trials, longer duration of statin therapy, retrospective analysis of neurosurgical procedures in patients with long term statins use, could be helpful for decisive evidence.

## Competing interests

All authors declare that they have no competing interests.

## Authors’ contributions

All authors participated in study design. KG and AK wrote the study protocol, and supervised all the stages of the research. KG managed supplying drug and placebo. AK performed surgeries. NM as a clinical pharmacist was involved in every steps of the project including literature review, writing the proposal, performing randomization and keeping it secret, collecting demographic data, design and filling the questionnaire, analyzing blood samples and providing article draft. SN and SA did neurological physical examination of patients, involved in blood sample collection, and assessed Karnofsky performance score. JS did all statistical analyses. MM and MJ consulted us in different stages of the project. KG, AK, JS, SA, and SN edited the draft. All authors read and approved the final manuscript.
